# Neonatal Perirhinal Lesions in Rhesus Macaques Alter Performance on Working Memory Tasks with High Proactive Interference

**DOI:** 10.3389/fnsys.2015.00179

**Published:** 2016-01-05

**Authors:** Alison R. Weiss, Ryhan Nadji, Jocelyne Bachevalier

**Affiliations:** ^1^Department of Psychology, Emory UniversityAtlanta, GA, USA; ^2^Division of Developmental Cognitive Neuroscience, Yerkes National Primate Research CenterAtlanta, GA, USA

**Keywords:** excitotoxic lesion, self-ordered task, serial order memory, perseveration, proactive interference

## Abstract

The lateral prefrontal cortex is known for its contribution to working memory (WM) processes in both humans and animals. Yet, recent studies indicate that the prefrontal cortex is part of a broader network of interconnected brain areas involved in WM. Within the medial temporal lobe (MTL) structures, the perirhinal cortex, which has extensive direct interactions with the lateral and orbital prefrontal cortex, is required to form active/flexible representations of familiar objects. However, its participation in WM processes has not be fully explored. The goal of this study was to assess the effects of neonatal perirhinal lesions on maintenance and monitoring WM processes. As adults, animals with neonatal perirhinal lesions and their matched controls were tested in three object-based (non-spatial) WM tasks that tapped different WM processing domains, e.g., maintenance only (Session-unique Delayed-nonmatching-to Sample, SU-DNMS), and maintenance and monitoring (Object-Self-Order, OBJ-SO; Serial Order Memory Task, SOMT). Neonatal perirhinal lesions transiently impaired the acquisition of SU-DNMS at a short (5 s) delay, but not when re-tested with a longer delay (30 s). The same neonatal lesions severely impacted acquisition of OBJ-SO task, and the impairment was characterized by a sharp increase in perseverative errors. By contrast, neonatal perirhinal lesion spared the ability to monitor the temporal order of items in WM as measured by the SOMT. Contrary to the SU-DNMS and OBJ-SO, which re-use the same stimuli across trials and thus produce proactive interference, the SOMT uses novel objects on each trial and is devoid of interference. Therefore, the impairment of monkeys with neonatal perirhinal lesions on SU-DNMS and OBJ-SO tasks is likely to be caused by an inability to solve working memory tasks with high proactive interference. The sparing of performance on the SOMT demonstrates that neonatal perirhinal lesions do not alter working memory processes *per se* but rather impact processes modulating impulse control and/or behavioral flexibility.

## Introduction

Working memory (WM) defines the psychological and neural processes responsible for keeping active a limited set of cognitive representations, and the executive capacity that acts upon those transiently stored representations. In other words, representations of objects, places, ideas, goals, or rules are maintained in WM and flexibly cooperate with process that monitor or manipulate the representations being kept “in mind.” Domain-specific models of WM have proposed that the lateral prefrontal cortex has a topographical organization according to specific WM processes. Evidence from human functional imaging (Ungerleider et al., 1998; D'Esposito et al., 1999; Owen et al., 1999; Petrides, 2000; Cannon et al., 2005), and lesion studies in monkeys (Mishkin et al., 1969; Passingham, 1975; Mishkin and Manning, 1978; Kowalska et al., 1991; Petrides, 1991, 1995), strongly support a distinction between the ventrolateral PFC (vlPFC) associated with maintenance processes and dorsolateral PFC (dlPFC) associated with monitoring/manipulation processes. However, more recent studies suggest that the prefrontal cortex is part of a broader network of interconnected brain areas involved in WM (see for review Constantinidis and Procyk, 2004). Specifically, medial temporal lobe (MTL) structures are also recruited during WM tasks (Kimble and Pribram, 1963; Diamond et al., 1989; Petrides, 1991, 1995, 2000; Davachi and Goldman-Rakic, 2001; Stern et al., 2001; Ranganath et al., 2004; Libby et al., 2012; Warren et al., 2012). In a recent report, Heuer and Bachevalier (2011) demonstrated that neonatal damage to the hippocampus in monkeys resulted in severe loss of WM-monitoring abilities, but spared WM-maintenance abilities. Given that the only direct inputs of the hippocampus to the PFC target the ventromedial PFC via the fornix, but not the vlPFC or dlPFC (Cavada et al., 2000; Croxson et al., 2005), bottom-up information from the hippocampus to the dlPFC will need to be realized via a multisynaptic pathway. Yet, the dlPFC projects back to the posterior hippocampus (Goldman-Rakic et al., 1984; Morris et al., 1999) providing a potential top-down mechanism regulating hippocampal-dependent WM processes.

Another MTL structure well positioned to play a prominent role in WM processes is the perirhinal cortex (PRh), which has direct reciprocal connections not only with the hippocampus but also with lateral and orbital PFC fields (Suzuki and Amaral, 1994; Lavenex et al., 2002; Saunders et al., 2005). In addition, electrophysiological and functional imaging studies have reported increased activity in PRh during object-based WM tasks, and PRh neurons of adult macaques are highly activated during WM tasks requiring the temporary maintenance of object representations (i.e., small-set delayed-match-to-sample). Such neuronal changes were not observed in other temporal visual areas, such as area TE (Lehky and Tanaka, 2007). Likewise, 2-deoxyglucose imaging studies indicate increased activity in PRh (but not the entorhinal cortex) during a delayed object alternation task; a task requiring the maintenance and monitoring of information in WM (Davachi and Goldman-Rakic, 2001). Taken together, these results point to a unique contribution of the PRh to performance on tasks that require the active/flexible representation of familiar objects.

Although the critical contribution of the PRh to recognition and stimulus-stimulus association memory has been well documented (Murray et al., 1993; Brown and Aggleton, 2001; Lavenex et al., 2004; Lee et al., 2006; Warburton and Brown, 2010), its participation in WM processes remains to be fully investigated. In a longitudinal developmental study aimed at tracking the long-term effects of neonatal PRh cortex lesions on memory processes, we recently demonstrated that these early-onset lesions yielded severe recognition memory deficits that emerged in infancy and persisted until adulthood (Zeamer et al., 2015; Weiss and Bachevalier, 2016). In the present study, we tested whether the same neonatal PRh lesions will result in WM deficits and whether the deficits will encompass both maintenance and monitoring WM processes. As they reached adulthood, animals with neonatal PRh lesions and their controls were successively tested in three object-based working memory tasks previously used to assess the effects of neonatal hippocampal lesions on WM processes (Heuer and Bachevalier, 2011, 2013).

## Material and methods

### Subjects

Fifteen adult rhesus macaques (*Macaca mulatta*), nine females and six males, participated in this study. Between postnatal days 10–12, the animals underwent surgery to create bilateral lesions of the perirhinal cortex, or sham operations. Six infant monkeys (three females, three males) were given MRI-guided ibotenic acid injections into perirhinal areas 35 and 36 (Group Neo-PRh), seven monkeys (five female, two male) underwent the same surgical procedures withholding any injections (Group Neo-C), and two additional monkeys (one female, one male) served as un-operated controls. At the time of this study, all animals were 6–7 years old and housed individually in a room with a 12 h light/dark cycle (7 AM/PM). Monkeys were fed Purina Old World Primate chow (formula 5047) and supplemented with fresh fruit enrichment. During behavioral testing, chow was restricted and the weight of the animals was monitored and maintained at or above 85% of the full feed weight. Water was given *ad libitum*. One cohort of subjects were born at the YNPRC breeding colony (Lawrenceville, Georgia), and a second cohort were born at the breeding colony of the University of Texas, M.D. Anderson Cancer Center Science Park (Bastrop, TX). At both institutions, all animals received similar rearing and behavioral procedures, including social interactions with age-matched peers and human caregivers as described previously (for detailed description see Goursaud and Bachevalier, 2007; Raper et al., 2013).

All animals had received extensive, but similar, cognitive testing before they participated in this experiment, including tests of incidental recognition memory (visual paired comparison at 1, 6, and 18 months; Zeamer et al., 2015), oddity learning (3 and 15 months), concurrent discrimination learning with devaluation (48 months), and object and spatial recognition memory (60 months; Weiss and Bachevalier, 2016).

All protocols were approved by the Institutional Animal Care and Use Committee at Emory University in Atlanta, Georgia and conformed to the NIH Guide for the care and use of Laboratory Animals (National Research Council (US), 2011).

### Neuroimaging and surgical procedures

All neuroimaging and surgical procedures were described in detail by Zeamer et al. (2015) and are briefly summarized below. To determine injection coordinates prior to surgical procedures and assess lesion extent post-surgery, subjects were given MRIs immediately prior to surgery and 6–8 days post-surgery. At both time points, animals were sedated (10 mg/kg of 7:3 Ketamine Hydrochloride, 100 mg/ml, and Xylazine, 20 mg/ml, administered i.m.) and intubated to allow inhalation of isoflurane (1–2%, v/v) and maintain an appropriate plane of anesthesia during the duration of the scan. An IV drip (0.45% NaCl and dextrose) was provided for normal hydration and the animal's head was restrained in a stereotaxic apparatus. Vital signs (heart and respiration rates, blood pressure, body temperature, and expired CO_2_) were constantly monitored during the scan and surgical procedures. The brain was imaged with a 3T Siemens Magnetom Trio system (Siemens Medical Solutions, Malvern, PA at YNPRC) using a 5-cm surface coil and two sets of images were obtained: (1) high-resolution structural images [3D T1-weighted fast spoiled gradient (FSPGR)-echo sequence, TE = 2.6 ms, TR = 10.2 ms, 25° flip angle, contiguous 1 mm sections, 12 cm FOV, 256 × 256 matrix]; and (2) Fluid Attenuated Inversion Recovery (FLAIR) images [TE = 140 ms, TR = 1000 ms, inversion time (TI) = 2200 ms, contiguous 3 mm sections, 12 cm FOV, 256 × 256 matrix; image sequences acquired in three series offset 1 mm posterior]. The pre-surgical T1-weighed images were used to calculate the injection sites and all pre- and post-surgical images were used to estimate the extent of PRh damage as well as damage to adjacent structures.

Following the pre-surgical scans, animals were maintained with Isoflurane gas (1–2%, v/v, to effect) during the surgical procedures, which were performed under deep anesthesia using aseptic conditions. The scalp was shaved and cleaned with chlorhexidine diacetate (Nolvasan, Pfizer). A long-lasting local anesthetic, Bupivacaine Hydrochloride (Marcaine 25%, 1.5 ml), was injected along the planned midline incision of the scalp, which extended from the occipital to the orbital ridge. After retraction of the galea, bilateral craniotomies (1 cm wide × 2.5 cm long) were made with an electric drill above the areas to be injected, and bone wax (Ethicon, Inc., Somerville, NJ; 2.5 g size) was applied as necessary to prevent bleeding. The dura was opened and injections of 0.4 μl ibotenic acid (Biosearch Technologies, Novato, CA, 10 mg/ml in PBS, pH 7.4, at a rate of 0.4 μl/min) were made 2 mm apart along the rostral-caudal length of the perirhinal cortex bilaterally. Sham-operated controls (Neo-C) underwent the same procedures, however once the dura was cut, no injections were made.

The dura, galea, and skin were closed in anatomical layers and the animal was removed from isoflurane, extubated, and closely monitored until complete recovery from anesthesia. Analgesic (acetaminophen, 10 mg/kg, p.o.) was given QID for 3 days after surgery. Additionally, animals received dexamethazone sodium phosphate (0.4 mg/kg, i.m.) to reduce edema, and Cephazolin (25 mg/kg, i.m.) once a day starting 12 h prior to surgery and ending 7 days after to prevent infection.

### Lesion assessment

Histological evaluations are unavailable, as all animals are currently participating in other experiments. Hence, lesion extent was estimated using the MRI images following methods described in details in earlier publications (Málková et al., 2001; Nemanic et al., 2002). Briefly, coronal FLAIR images acquired 1-week post-surgery were used to examine areas with water hyper-signals (edema) induced by cell death. Areas of hyper-signals seen in each coronal section were drawn onto corresponding coronal sections of a normal 1-week-old rhesus monkey brain (J. Bachevalier, unpublished atlas) using Adobe Photoshop. These images were then imported into Image J® and the surface area of hyper-signals in brain regions of interest (PRh, visual area TE/TEO, entorhinal cortex, parahippocampal cortex, amygdala, and hippocampus) was calculated in pixels^2^ and multiplied by image thickness (1 mm) to obtain the lesion volume. The percent of damage to each structure was obtained by dividing the volume of the lesion for a given structure by the volume of that same structure in the control atlas and multiplying by 100.

### Apparatus and stimuli

All behavioral tasks were conducted using the Wisconsin General Testing Apparatus (WGTA) located in a dark room with a white-noise generator. Monkeys were transferred from their home cages and positioned in the WGTA facing a tray with 3 recessed food wells (2 cm diameter, 1 cm deep, spaced 13 cm apart). Correct responses were rewarded with preferred food rewards (i.e., mini-marshmallow, jelly bean, M&M etc.)

### Session-unique delayed nonmatching-to-sample (SU-DNMS)

Session-Unique Delayed Nonmatching-to-Sample (SU-DNMS) measured the maintenance of information in working memory and used training procedures described in Heuer and Bachevalier (2011). For each daily training session, a new pair of objects was selected from a collection of 1000 junk objects without replacement. Each trial consisted of two phases: sample and choice. During the sample phase, the monkey was presented with a single object covering a reward, followed by a delay of 5 s. In the choice phase, two objects, the sample object and the second object, were presented and the monkey was rewarded for selecting the object that was not rewarded during the sample phase. Following a 30 s intertrial interval, the same two objects were used for the next trial as well as for all 30 trials of the daily session. The object serving in the sample phase varied on each trial using a pseudorandom sequence. In the first trial, the two objects were novel, but as the daily session progresses, the two stimuli became highly familiar and generated proactive interference. Thus, in SU-DNMS familiarity/novelty judgments cannot be used to guide responses, rather subjects were required to generate responses based on recency memory and inhibit responses based on recognition memory. Learning criterion was set at 90% or better (27 out of 30) in one session, followed by a performance of 80% or better (24 out of 30) in the next training session. Training was discontinued after a maximum of 1000 trials if criterion was not met. Once subjects met learning criterion at the 5 s delay, testing was continued in the same way using a 30 s delay and a 30 s inter-trial interval. At this longer delay, subjects performed 20 trials per day, again using a novel pair of objects each day, until a learning criterion of 85% averaged over two consecutive testing sessions was achieved, or to a maximum of 500 trials.

The total number of errors (incorrect choices) until meeting criterion at each delay was used as a measure of learning. We also examined how the errors were distributed between the two objects across the daily trials. If errors were distributed equally between the objects, it suggested that the cause of the errors was an impaired ability to maintain information in working memory. On the other hand, if errors were biased toward one object, it instead suggested that the cause of the errors was an impairment of non-mnemonic processes important to support task performance. To test this proposal, we computed an Object Error Distribution Ratio by calculating the absolute value of percent errors made for each object during each daily session minus 50% [# Errors per Object/Total Errors in Session)^*^100%)−50%)]. These values ranged from 0–50, where 0 represented an equal distribution of errors between the two objects and 50 represented a complete bias toward one of the objects.

### Object self-ordered task (OBJ-SO)

This task measured both maintenance and monitoring WM processes, and procedures replicated those described in Heuer and Bachevalier (2011). A set of three new objects, not used in the SU-DNMS task, were selected for the OBJ-SO task. During each daily testing session, monkeys chose three objects, one at a time, during three successive trials. At the start, all three objects were presented covering each of the three food wells with a food reward (Trial 1). Once the monkey made a first choice, the position of the objects on the tray was shuffled and only the two objects unselected in Trial 1 were baited in Trial 2. After the second choice, the positions of the objects were once again shuffled and only the single remaining (unselected) object in Trials 1 and 2 was baited on Trial 3. The same three objects were used in all daily testing sessions and were presented at 10 s inter-trial intervals. If, at any time during Trial 2 or 3, the monkey selected an unbaited object, this initial error was scored as a primary error and a correction procedure was initiated. Correction procedures involved reordering the objects and re-presenting them to the monkey until a rewarded object was selected. The number of times the correction procedure was repeated indicated the number of perseverative errors. For analyses, primary and perseverative errors were calculated separately for Trial 2 or Trial 3. Additionally, the percent of errors on Trial 3 that were “repeats” of the errors made on Trial 2 were also tabulated as a measure of impulsive responding.

Learning criterion for the OBJ-SO task was met when subjects scored 85% correct across 10 consecutive daily sessions (three primary errors or fewer), or testing was discontinued if subjects reached a maximum of 50 daily sessions. Thus, in OBJ-SO monkeys were rewarded for making choices based on the temporal sequence of their own object selections in previous trials of the daily testing session.

### Serial order memory task (SOMT)

Similar to the OBJ-SO task, the SOMT assessed both maintenance and monitoring WM processes and was delivered using procedures described by Heuer and Bachevalier (2013). A pool of new objects was selected for each trial of this task from another collection of 1000 junk objects that differed in size, shape, color, and texture. The objects were divided in 25 bins of 40 objects each and each bin was selected for testing one at a time until all 25 bins were used before re-using the first bin. Thus, objects only reappeared about once per month. A trial of SOMT consisted of two phases: the sample phase and the test phase. In the sample phase, a list of objects were presented one at a time at 10 s intervals covering the baited center food-well. After displacing the last object of the list and retrieving the food rewards, there was a 10 s delay after which the test phase began. In the test phase, two of the objects from the list were selected and covered the lateral food-wells. The monkey was rewarded for displacing the object that occurred earliest in the list. After a 30 s inter-trial interval, the next trial began using a new set of objects. A total of 10 trials were given for each daily session.

The monkeys were first trained to criterion using lists of three objects. Training progressed in stages: during Stage 1, the test phase paired the first and third objects (1v3), Stage 2 paired the first and second (1v2), and Stage 3 paired the second and third (2v3). The monkey was required to score 80% (8/10) correct during a daily session before moving to the next stage. If the monkey scored 70% (7/10), then that stage was repeated the following session. If the monkey scored 60% or less (6/10), then they were moved back to the previous stage. Once the monkey completed the three-object version, they moved on to a four-object version including six stages in which the orders of object pairings in the test phase were as follows: 1v4, 1v3, 1v2, 2v4, 3v4, and 2v3. It is worth noting that only discrimination problems including objects 2v3 required the animals to maintain the order of the objects presented in the list, since with training monkeys could learn that for the other discrimination problems Objects 1 were always rewarded and Objects 4 were never rewarded. After completing training on the four-object SOMT, monkeys were tested with probe trials.

Probe trials were administered to assess the ability of the monkeys to track the serial position of objects presented in sequence. This training was identical to the four-object version described above, except that half of the trials (five trials) were judgments between 1v4, and the other half (five trials) were judgments between 2v3. These two trial types were randomized within a daily session so that the monkey could not anticipate which temporal judgments would occur on each trial. Probe trials, therefore, required the monkeys to track ALL of the stimuli in the list. Ten probe trials were administered daily for three consecutive days, resulting in a total of 15 trials of each type. A ratio score was calculated by dividing the total number of correct responses on “inner” pairings (2v3 trials) by the total number of correct responses on “outer” pairings (1v4 trials). A ratio score above or below 1 indicated superior performance on one type of temporal discrimination over another, whereas a score equal to 1 indicated equivalent performance on both trial types.

### Data analyses

Scores of the control animals from the Texas cohort (*n* = 5) and control animals of the Georgia cohort (*n* = 4; see Subjects) were compared across all measures using independent sample *t*-tests. None reached significance, and so these groups were collapsed in a single control group for all subsequent analyses.

Data obtained from SU-DNMS and OBJ-SO followed a normal distribution, and so repeated measures ANOVAs were used to compare the scores of the Neo-PRh and Neo-C groups. For SU-DNMS, 2 × 2 ANOVAs (Group × Delay 5–30 s) using Delay as the repeated-factor were performed on the two parameters (errors to reach criterion, object error distribution ratio). For OBJ-SO, primary and perseverative Errors were analyzed with a Three-way ANOVA (Group × Error Type × Trial) with repeated measures for the last two factors. Finally, independent sample *t*-tests were used for both tasks to compare the performance of Neo-PRh and Neo-C groups on each measure.

Data from SOMT did not follow a normal distribution, with the exception of the Inner:Outer ratio score. Both nonparametric and parametric analyses were used for all measures. Given the similar pattern of results obtained with both analyses, only the parametric tests will be reported in the “Results” section below. For number of sessions to criterion, a 2 × 2 ANOVA (Group × Object-Pairing) with repeated measures for the second factor was performed. When sphericity was violated, degrees of freedom were adjusted using the Greenhouse-Geisser correction. Finally, group differences on probe trials (Inner:Outer ratio) were assessed using an independent sample *t*-test.

Correlations between extent of neonatal PRh lesions or unintended damage to adjacent areas and scores on the three tasks were performed with Pearson correlation. Lastly, for all ANOVAs, effect sizes are reported using eta squared (η^2^) and calculated by dividing the sums of squares for the effect of interest by the total sums of squares (Cohen, 1973; Levine and Hullett, 2002; Keppel and Wickens, 2004). For all *T*-tests, effect sizes are reported using Cohen's *d* and calculated by dividing the difference between the means of the two groups by the pooled standard deviations (Rosnow and Rosenthal, 1996).

## Results

### Lesion assessment

**Figure 1:**
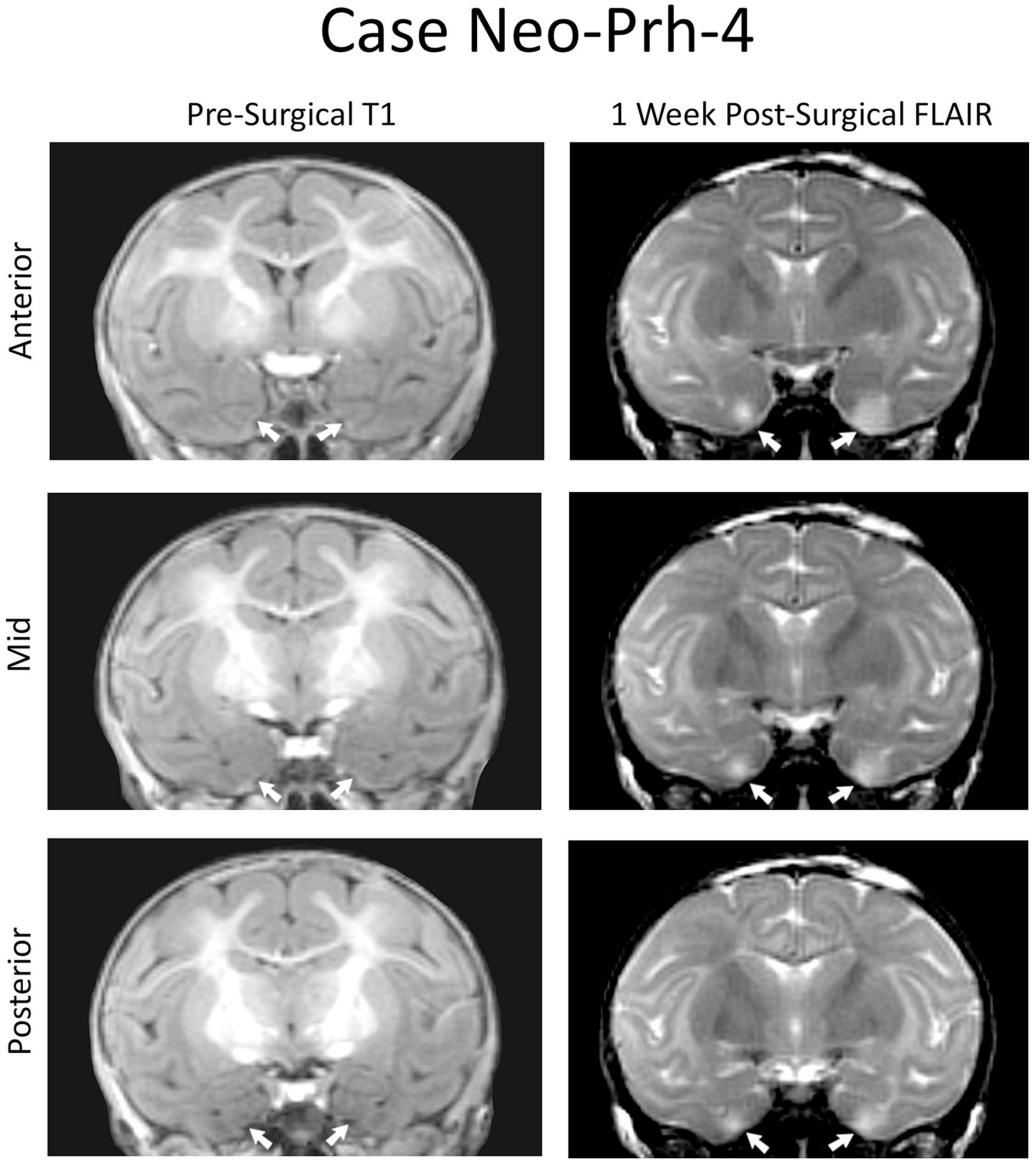
**Coronal MRI from a representative case (Neo-PRh-4)**. Pre-surgical structural T1-weighted images at three rostro-caudal levels through the perirhinal cortex (left column). Post-surgical FLAIR images (right column) at the same rostro-caudal levels show hypersignals (whiter areas) that are indicative of edema and cell damage. Arrows point to the rhinal sulcus on the left and to hypersignals on the right.

**Figure 2:**
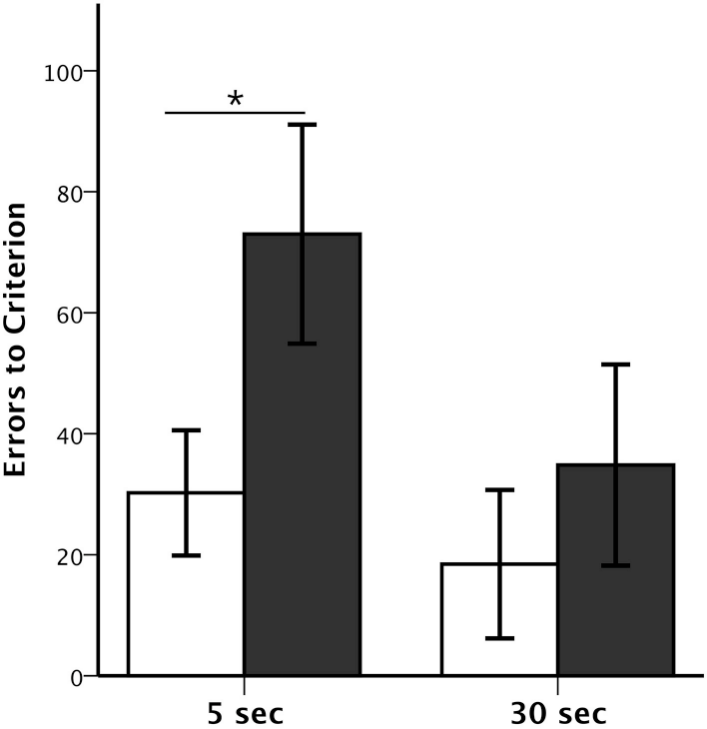
**Session-Unique DNMS performance**. Average number (±SEM) of errors to reach criterion on Session-Unique DNMS at delays of 5 and 30 s for animals with neonatal perirhinal lesions (filled bars) and controls (open bars). ^*^p < 0.05.

Detailed lesion assessments for all Neo-PRh animals have been published in Zeamer et al. (2015) and percentage of damage to the PRh and adjacent structures is given for each subject of Group Neo-PRh in Table 1. Briefly, all Neo-PRh animals received extensive bilateral damage to the PRh, averaging 73.6% (min = 67.1%, max = 83.3%). Unintended damage occurred in all cases, mostly in the entorhinal cortex (ERh) (average = 20.6%, min = 5.4%, max = 34.5%), but also minimally in area TE (average = 2.5%, min = 0.1%, max = 7.11%). Four of the six Neo-PRh subjects had negligible damage to the anterior hippocampus (average = 0.8%), and three of the six subjects had minimal damage to the amygdala (average = 2.5%). The PRh lesion of a representative case (Neo-PRh-4) is illustrated in Figure 1 and two additional cases can be seen in previous publications (see Zeamer et al., 2015, see Figure 2 for case Neo-PRh 3 and Weiss and Bachevalier, 2016, see Figure 1 for case Neo-PRh-2).

**Table 1:** **Extent of neonatal perirhinal lesions**.

**Subjects**	**PRh**				**ERh**			
	**L%**	**R%**	**X%**	**W%**	**L%**	**R%**	**X%**	**W%**
Neo-PRh-1	89.76	79.91	83.34	69.04	28.51	2.28	15.39	0.65
Neo-PRh-2	68.16	70.58	69.37	48.11	17.72	20.65	19.19	3.36
Neo-PRh-3	65.45	81.02	73.23	53.02	7.72	3.12	5.42	0.24
Neo-PRh-4	59.40	74.73	67.06	44.39	11.55	17.84	14.69	2.06
Neo-PRh-5	75.90	66.81	71.35	50.71	38.60	29.86	34.32	11.53
Neo-PRh-6	74.12	80.31	77.22	59.53	25.34	43.64	34.49	11.06
Average	72.13	75.06	73.60	54.13	21.57	19.57	20.57	4.87

L%, percent damage to left hemisphere; R%, percent damage to right hemisphere; X%, average damage to both hemispheres; W%, weighted damage to both hemispheres [W% = (L% × R%)/100]. PRh, perirhinal cortex; ERh, entorhinal cortex. Lesion extents from cases Neo-PRh-1 thru Neo-PRh-6 were previously reported in Zeamer et al. (2015).

### SU-DNMS

The numbers of trials and errors to reach the learning criterion at each delay, 5 and 30 s, as well as the Object Error Distribution Ratios are reported in Table 2. All animals reached criterion at both the short and long delays, although animals with Neo-PRh lesions made twice as many errors (Mean: 73 at 5 s delay and 34.8 at 30 s delay) than controls (Mean: 30.2 at 5 s delay and 18.4 at 30 s delay; see Figure 2). These group differences were confirmed by a significant group effect on the number of errors to reach criterion [*F*_(1, 13)_ = 5.156, *p* = 0.041, η^2^ = 0.28]. Planned comparisons revealed that the group difference at the 5 s delay was significant [*t*_(13)_ = 2.207, *p* = 0.046, *d* = 1.12], but not at the 30 s delay [*t*_(13)_ = −0.811, *p* = 0.432, *d* = 0.42]. Furthermore, although both groups improved their performance from the 5 to the 30 s delay (see Figure 2), the delay effect and the interaction (Group × Delay) were not reliable [*F*_(1, 13)_ = 2.803, *p* = 0.118, η^2^ = 0.14; *F*_(1, 13)_ = 0.783, *p* = 0.392, η^2^ = 0.05], indicating that the magnitude of improvement was similar for both groups.

**Table 2:** **Performance on the SU-DNMS and Obj-SO tasks**.

**Groups**	**SU-DNMS**						**OBJ-SO**				
	**Trials to criterion**		**Errors to criterion**		**Object error distribution ratio**		**Sessions to criterion**	**Primary errors**		**Perseverative errors**	
	**5 s**	**30 s**	**5 s**	**30 s**	**5 s**	**30 s**		**Trial 2**	**Trial 3**	**Trial 2**	**Trial 3**
**Neo-PRh**											
Neo-PRh-1	360	0	106	0	22.6	0.0	50	23	33	8	57
Neo-PRh-2	90	360	30	110	12.8	24.6	50	24	36	11	53
Neo-PRh-3	420	160	102	52	25.8	32.9	50	16	26	4	35
Neo-PRh-4	480	60	129	12	20.1	8.9	50	16	31	7	59
Neo-PRh-5	180	80	43	20	25.8	25.4	8	1	4	0	2
Neo-PRh-6	90	60	28	15	15.9	18.3	50	11	32	3	64
Average	270.0	120.0	73.0	34.8	20.5	18.4	43.0	15.2	27.0	5.50	45.00
**Neo-C**											
Neo-C-1	0	0	0	0	0.0	0.0	0	0	0	0	0
Neo-C-2	–	–	–	–	–	–	–	–	–	–	–
Neo-C-3	150	320	35	114	17.5	27.2	6	2	8	0	16
Neo-C-4	240	80	68	16	9.5	22.9	11	2	5	0	13
Neo-C-5	120	0	26	0	18.5	0.0	5	4	3	1	0
Neo-C-6	0	0	0	0	0.0	0.0	1	1	0	0	0
Neo-C-7	300	0	71	0	14.9	0.0	26	6	15	1	17
Neo-C-8	–	–	–	–	–	–	–	–	–	–	–
Neo-C-9	0	0	0	0	0.0	0.0	15	7	13	1	4
Neo-C-10	270	60	66	18	18.1	23.6	50	13	41	4	62
Neo-C-11	30	80	6	18	16.7	23.3	0	0	0	0	0
Average	123.3	60.0	30.2	18.4	10.6	10.8	12.7	3.9	9.4	0.8	12.4
**Neo-H**											
Neo-H-1	0	220	0	55	0	15.2	50	8	27	1	28
Neo-H-2	30	40	4	11	25.0	33.3	50	13	33	11	52
Neo-H-3	570	40	190	17	13.2	14.6	0	0	0	0	0
Neo-H-4	60	20	9	10	35.7	10.0	50	15	28	3	39
Neo-H-5	330	0	91	0	16.8	0.0	50	17	32	3	39
Neo-H-6	30	0	5	0	10.0	7.1	50	8	26	2	34
Average	170.0	53.3	49.8	15.5	16.8	13.4	41.7	10.2	24.3	3.3	32.0

For Session Unique Delayed Non-Match to Sample (SU-DNMS), scores are number of trials and errors to criterion and the error distribution ratio at each delay. For the Object Self-Ordered task (OBJ-SO), scores are number of sessions and errors to criterion. Neo-C-2 and Neo-C-8 were not tested on SU-DNMS or OBJ-SO. Data from Neo-C-1 thru Neo-C-6 and Neo-C-11 previously reported in Heuer and Bachevalier (2011). Data Neo-H-1 thru Neo-H-6 used for comparison in Section Comparisons with Neonatal Hippocampal Lesions and also reported in Heuer and Bachevalier (2011).

The Object Error Distribution Ratio (Table 2) was also higher in animals with Neo-PRh lesions than controls at both delays, indicating a tendency to preferentially select one object over the other [*F*_(1, 13)_ = 3.782, *p* = 0.075, η^2^ = 0.23]. Neither the delay effect nor the interactions between the two factors reached significance [*F*_(1, 13)_ = 0.100, *p* = 0.756, η^2^ = 0.01 and *F*_(1, 13)_ = 0.150, *p* = 0.705, η^2^ = 0.01, respectively]. Yet, planned comparisons indicated that the group difference was significant at the 5 s delay but not at the 30 s delay [*t*_(13)_ = 2.561, *p* = 0.024, *d* = 1.42 and *t*_(13)_ = 1.143, *p* = 0.273, *d* = 0.61, respectively].

Additionally, errors made during the first block of 10 trials and last bock of 10 trials in each daily session of the SU-DNMS task were tallied separately to determine if the monkeys tended to make more errors at the end of the session. A Group × Trial-Block (first-last) ANOVA with repeated measure for the second factor revealed a significant main effect of Group at the 5 s delay [*F*_(1, 13)_ = 5.107, *p* = 0.042, η^2^ = 0.282], but not at the 30 s delay [*F*_(1, 13)_ = 0.754, *p* = 0.401, η^2^ = 0.055] and a significant effect of Trial-Block at the 5 s delay [*F*_(1, 13)_ = 5.084, *p* = 0.042, η^2^ = 0.272] but not at the 30 s delay *F*_(1, 13)_ = 3.672, *p* = 0.078, η^2^ = 0.218]. None of the interactions were significant [5 s: *F*_(1, 13)_ = 0.640, *p* = 0.438, η^2^ = 0.034; 30 s: *F*_(1, 13)_ = 0.142, *p* = 0.712, η^2^ = 0.008]. Thus, both groups of monkeys tended to make more errors on the last 10 trials than on the first 10 trials at 5 s delay, but not at 30 s delay.

### OBJ-SO

**Figure 3:**
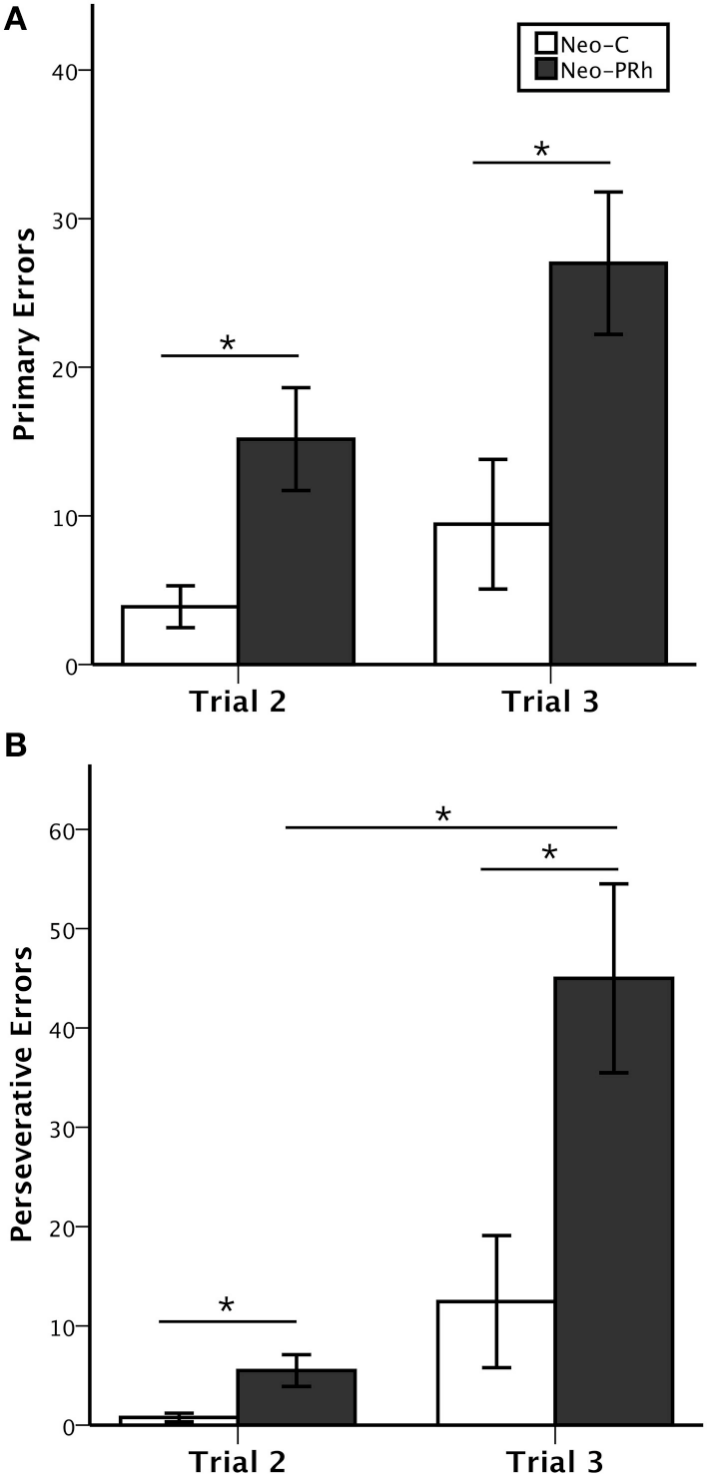
**Object self-ordered task performance**. Average number (±SEM) of primary errors **(A)** and perseverative errors **(B)** to criterion on the object self-ordered task (Obj-SO) at delays of 5 and 30 s for animals with neonatal perirhinal lesions (filled bars) and controls (open bars). ^*^p < 0.05.

Control animals reached criterion in an average of 12.7 testing days. In contrast, all but one of the six animals with Neo-PRh cortex lesions (Neo-PRh-5) failed to reach criterion within the limit of testing (50 testing days), resulting in an averaged group performance of 43 [*t*_(13)_ = −3.454, *p* = 0.004, *d* = 1.81; see Table 1]. As shown in Figures 3A,B, this learning impairment was also reflected by a greater number of primary and perseverative errors on Trial 2 and Trial 3 made by Neo-PRh animals as compared to the Neo-C animals [Primary errors: *t*_(13)_ = −3.444, *p* = 0.004, *d* = 1.68 and *t*_(13)_ = −2.647, *p* = 0.020, *d* = 1.41 for Trial 2 and Trial 3, respectively; Perseverative errors: *t*_(5.736)_ = −2.836, *p* = 0.031, *d* = 1.61 and *t*_(13)_ = −2.901, *p* = 0.012, *d* = 1.50, for Trial 2 and Trial 3 respectively].

The Three-way ANOVA (Group × Error types × Trials) revealed significant main effects of Group [*F*_(1, 13)_ = 9.597, *p* = 0.008, η^2^ = 0.42] and Trial [*F*_(1, 13)_ = 22.716, *p* < 0.001, η^2^ = 0.55], but not of Error Type [*F*_(1, 13)_ = 2.819, *p* = 0.117, η^2^ = 0.15]. The Three-way interaction also reached significance [*F*_(1, 13)_ = 10.545, *p* = 0.006, η^2^ = 0.21]. Thus, although both groups made more primary and perseverative errors on Trial 3 than on Trial 2, Group Neo-C had a similar increase in primary and perseverative errors across trials. By contrast, for Group PRh, the increase in perseverative errors from Trial 2 to Trial 3 was greater in magnitude than the increase in primary errors [Group × Trial interaction: *F*_(1, 13)_ = 7.217, *p* = 0.019, η^2^ = 0.13 and *F*_(1, 13)_ = 2.172, *p* = 0.164, η^2^ = 0.07, for Perseverative and Primary Errors, respectively].

Finally, to determine whether the increase of errors in animals with Neo-PRh lesions was due to impulsive reactivity, we assessed the animals' tendency to select in Trial 3 the same incorrect object they selected in Trial 2. The percent of errors on Trial 3 that repeated the errors on Trial 2 did not significantly differ between groups [*t*_(13)_ = −0.435, *p* = 0.671, *d* = 0.24].

### SOMT

The numbers of sessions to reach criterion at each stage of object pairings on the three-Object and four-Object versions of this task are reported in Table 3. All monkeys acquired the task within the maximum number of sessions (20 per stage). On the three-Object version, the effects of group (Neo-C vs. Neo-PRh), Object-Pairing stages (i.e., 1v3, 1v2, 2v3) and their interaction did not reach significance [*F*_(1, 12)_ = 0.827, *p* = 0.381, η^2^ = 0.064; *F*_(1.230, 14.758)_ = 3.312, *p* = 0.083, η^2^ = 0.216; *F*_(1.230, 14.758)_ = 0.023, *p* = 0.920, η^2^ = 0.002, respectively]. A similar pattern emerged on the four-Object version [Group: *F*_(1, 12)_ = 3.197, *p* = 0.099, η^2^ = 0.210; six Object-Paring stages: *F*_(2.503, 30.040)_ = 0.490, *p* = 0.659, η^2^ = 0.036; Group × Object-Pairing interaction: *F*_(2.503, 30.040)_ = 1.007, *p* = 0.392, η^2^ = 0.075]. Therefore, both groups performed similarly on the three-Object and four-Object versions of the task.

**Table 3:** **Performance on the SOMT task**.

**Groups**	**SOMT 3-Object**			**SOMT 4-Object**						***SOMT Probe***
	**1v3**	**1v2**	**2v3**	**1v4**	**1v3**	**1v2**	**2v4**	**3v4**	**2v3**	**Inner:Outer Ratio**
**Neo-PRh**										
Neo-PRh-1	2	7	1	3	1	1	2	3	1	0.62
Neo-PRh-2	3	3	1	3	3	2	1	2	6	0.71
Neo-PRh-3	2	5	1	1	2	1	1	1	2	0.83
Neo-PRh-4	1	4	7	5	2	5	2	1	1	1.08
Neo-PRh-5	1	11	3	2	5	1	1	1	6	1.00
Neo-PRh-6	2	1	4	1	1	1	3	8	1	0.79
Average	1.8	5.2	2.8	2.5	2.3	1.8	1.7	2.7	2.8	0.84
**Neo-C**										
Neo-C-1	1	3	1	2	2	9	1	1	1	1.08
Neo-C-2	–	–	–	–	–	–	–	–	–	–
Neo-C-3	1	2	3	1	1	3	1	1	1	0.93
Neo-C-4	1	18	1	1	2	2	2	1	1	1.00
Neo-C-5	1	2	2	1	1	3	1	1	1	1.20
Neo-C-6	1	1	3	1	2	3	1	1	1	1.00
Neo-C-7	2	1	1	2	3	2	1	2	4	0.87
Neo-C-8	–	–	–	–	–	–	–	–	–	–
Neo-C-9	1	2	4	2	2	1	1	1	4	0.69
Neo-C-10	–	–	–	–	–	–	–	–	–	–
Neo-C-11	2	4	3	1	1	1	3	4	1	–
Average	1.1	4.1	2.1	1.4	1.9	3.3	1.1	1.1	1.9	0.97
**Neo-H**										
Neo-H-1	1	3	2	1	1	1	1	1	8	0.54
Neo-H-2	1	2	4	1	1	7	1	6	5	0.62
Neo-H-3	1	10	2	1	1	5	2	1	5	1.00
Neo-H-4	2	9	1	1	1	1	1	1	3	0.53
Neo-H-5	1	7	1	1	2	2	2	3	4	0.71
Neo-H-6	1	1	1	1	1	1	1	2	5	0.50
Average	1.2	5.3	1.8	1.0	1.2	2.8	1.3	2.3	5.0	0.65

Scores are the numbers of sessions to criterion for each of the object pairings in the 3-objects and 4-objects version of the Serial Order Memory Task (SOMT). Probe ratio are correct choices for “inner” (2v3) problems over correct choices for “outer” (1v4) problems. Neo-C-2, Neo-C-8, and Neo-C-10 were not tested on the SOMT, and Neo-C-11 was not given the SOMT Probe trials. Data from Neo-C-1 thru Neo-C-6 previously reported in Heuer and Bachevalier (2013). Data from animals Neo-H-1 thru Neo-H-6 used for comparison in Section Comparisons with Neonatal Hippocampal Lesions and also reported in Heuer and Bachevalier (2013).

Results of the probe trials are reported in Table 3. The Inner:Outer ratio scores of the Neo-PRh group averaged 0.84, indicating slightly better performance on 1v4 pairings that 2v3 pairings. The Neo-C group averaged 0.97, indicating approximately equal performance on both pairings. However, the group difference was not significant [*t*_(11)_ = −1.375, *p* = 0.197, *d* = 0.76].

### Correlations

Finally, none of the correlations between the average extent bilateral of PRh damage and scores on each of the three working memory tasks reached significance (all *p*s > 0.05), indicating that greater extent of lesions was not related to performance on any of the tasks (see Supplemental Materials for details).

### Comparisons with neonatal hippocampal lesions

To investigate how the pattern of deficits after the Neo-PRh lesions contrast with those previously reported after neonatal hippocampal (Neo-H) lesions, scores of Neo-PRh and Neo-C groups on the three working memory tasks were compared to those obtained by the Neo-H groups (Heuer and Bachevalier, 2011, 2013). As shown in Table 2, Neo-H lesions appear to affect SU-DNMS acquisition (50 and 16 errors for 5 and 30 s, respectively) to a smaller degree than Neo-PRh lesions (73 and 35 errors for 5 and 30 s respectively). However, differences between the three groups did not reach significance [5 s errors: *F*_(2, 20)_ = 1.262, *p* = 0.307, η^2^ = 0.123; 30 s errors: *F*_(2, 20)_ = 0.574, *p* = 0.573, η^2^ = 0.060]. In contrast, the Neo-PRh group was equally impaired in learning the OBJ-SO task as the Neo-H group (see Table 2), both groups averaging 43 and 44 sessions to reach criterion, respectively, as compared to 13 sessions for the controls, [*F*_(2, 20)_ = 7.164, *p* = 0.005, η^2^ = 0.443; Neo-PRh vs. Neo-H: *t*_(18)_ = 0.130, *p* = 0.898, *d* = 0.070; Neo-PRh vs. Neo-C: *t*_(18)_ = 3.236, *p* = 0.005, *d* = 1.810; Neo-H vs. Neo-C: *t*_(18)_ = −3.094, *p* = 0.006, *d* = 1.568]. Finally, comparisons between the effects of Neo-H lesions and Neo-PRh lesions on the SOMT (Table 3) indicated that the Neo-H group required more sessions (five sessions) to complete the 2v3 phase of the four-Object version than the Neo-PRh group (three sessions) or controls (one session) [*F*_(2, 19)_ = 5.336, *p* = 0.016, η^2^ = 0.386; Neo-PRh vs. Neo-H: *t*_(17)_ = −2.026, *p* = 0.059, *d* = 1.025; Neo-PRh vs. Neo-C: *t*_(17)_ = 1.083, *p* = 0.294, *d* = 0.537; Neo-H vs. Neo-C: *t*_(17)_ = −3.249, *p* = 0.005, *d* = 2.114]. This impairment of temporal order memory for the inner items of a list by the Neo-H group was also apparent in Probe trials, where Neo-H monkeys had lower Inner:Outer ratios (0.68) than the Neo-PRh monkeys (0.84) or Controls (0.97) [*F*_(2, 18)_ = 5.350, *p* = 0.017, η^2^ = 0.401; Neo-PRh vs. Neo-H: *t*_(16)_ = 1.870, *p* = 0.080, *d* = 1.038; Neo-PRh vs. Neo-C: *t*_(16)_ = −1.324, *p* = 0.204, *d* = 0.757; Neo-H vs. Neo-C: *t*_(16)_ = −3.265, *p* = 0.005, *d* = 1.806].

## Discussion

This study investigated the effects of neonatal PRh-lesions on WM processes when animals reached adulthood. The results indicate that neonatal PRh-lesions slightly, but only transiently, impaired WM maintenance processes measured by the SU-DNMS task and impaired WM maintenance/monitoring processes measured by the OBJ-SO task. In contrast to both SU-DNMS and OBJ-SO tasks that generated high proactive interference, performance on the SOMT that was devoid of proactive interference was not altered by the neonatal PRh lesions. The results suggest that neonatal PRh lesions may impact the ability to resolve proactive interference and/or inhibit perseverative responding rather than affecting working memory processes *per se*. These findings will be discussed in turn.

### Maintenance

Monkeys with Neo-PRh lesions initially learned SU-DNMS more slowly than controls. However, the mild impairment at the short delay was not evident with further training at the longer delay of 30 s. The same groups of animals were tested on several other memory tasks from infancy through adulthood, and their performance on these tasks can help us reject several interpretations of the transient impairment in the SU-DNMS task. For example, animals with neonatal PRh lesions did not differ from controls in learning a trial-unique delayed nonmatching task indicating no significant impact of the Neo-PRh lesions on perceptual abilities, formation of object representation, learning reward contingencies, or motivation to perform a task (Weiss and Bachevalier, 2016). Furthermore, the impairment at the 5 s of the SU-DNMS could not be explained by an inability to maintain object representation across the short delay, given the normal performance at the longer delay of 30 s. However, one distinct feature of the SU-DNMS task that has not been addressed with prior memory tasks given to these groups of animals, but that could be relevant to their impairment in the SU-DNMS, is the increased interference encountered by the animals while responding to successive trials. Indeed, in contrast to all other memory tasks previously performed by the animals, SU-DNMS uses the same two stimuli on every trial of a daily session, generating increased proactive interference as the animals progressed through the task. Thus, the learning impairment observed in animals with Neo-PRh lesions at the 5 s delay could be the result of difficulties learning to resolve or inhibit interference. Interestingly, the mild and transitory impairment of the Neo-PRh subjects during the SU-DNMS task is reminiscent to that reported earlier by Eacott and colleagues after rhinal (perirhinal and entorhinal) cortex lesions in adulthood (Eacott et al., 1994). In this latter study, adult monkeys with rhinal lesions were tested in a matching-to-sample task using four stimuli and showed transient impairment especially at the shortest delays used and not at the longer delays, and then performed normally when re-tested with only two stimuli. This similar pattern of transient deficits after the early-onset and late-onset lesions suggests very little recovery of SU-DNMS performance after the early-onset PRh lesions.

A large body of work has already demonstrated that the hippocampus may be critical to reduce proactive interference (Shapiro and Olton, 1994; Butterly et al., 2012; but see Aggleton et al., 1986; Bachevalier et al., 2013). Given that the majority of sensory inputs reaching the hippocampus are relayed through the perirhinal cortex, the Neo-PRh lesions could have disconnected the hippocampus from receiving this flow of information and yielded decreased resistance to interference. However, this explanation seems implausible given that direct damage to the hippocampus does not impair performance on the SU-DNMS (Heuer and Bachevalier, 2011). An alternative explanation may relate to the important interconnections of the perirhinal cortex with the ventrolateral PFC (vlPFC) and orbital frontal cortex (OFC; Lavenex et al., 2002; Petrides and Pandya, 2002). Both vlPFC and OFC lesions in adult monkeys yield deficits in rule-learning that were attributed to perseverative interference generated from competition between well-established responses (Butter, 1969; Passingham, 1975; Mishkin and Manning, 1978; Dias et al., 1996; Meunier et al., 1997; Baxter et al., 2008, 2009). Furthermore, like performance of Neo-PRh monkeys, monkeys with vlPFC lesions require more trials than controls to acquire the DNMS rule and tend to make perseverative errors, but after learning the task, they perform normally on subsequent tests with longer delays (Kowalska et al., 1991). Monkeys with OFC lesions are similarly slow to acquire the DNMS rule, yet their deficit is not overcome with additional training (Meunier et al., 1997). Thus, the deficit in learning the SU-DNMS at short delay may have resulted from a disconnection of the vlPFC from the PRh, preventing vlPFC from accessing object-representations generated by PRh. Yet, the learning deficit in the SU-DNMS after the neonatal PRh lesions was only transitory as was the learning deficit following vlPFC lesions. This improvement in performance suggests that with further training, animals with such lesions can overcome or suppress their perseverative habits, presumably, by developing alternate strategies supported by other PFC areas, such as the OFC. A recent study investigating the effects of neonatal lesions to the vlPFC and OFC separately or in combination demonstrated that, in the absence of a functional vlPFC in infancy, the OFC can take over and support learning skills (Malkova et al., 2015).

### Monitoring

In comparison to the transient impairment on the WM maintenance task, SU-DNMS, the same neonatal PRh lesions severely impacted acquisition of the OBJ-SO task in all but one of the Neo-PRh monkeys. Furthermore, the source of errors during OBJ-SO acquisition differed between the Neo-PRh and Neo-C groups. The Neo-PRh group made more primary errors than the controls, but the increase in primary errors from Trial 2 to Trial 3 was similar for both groups. Furthermore, although the Neo-PRh group made also more perseverative errors than controls, the increase in perseverative errors from Trial 2 to Trial 3 was greater in magnitude for animals with Neo-PRh lesions than for controls. This pattern of results indicates that monkeys with neonatal PRh lesions may be unable to monitor the order of self-generated responses. Alternatively, like the mild learning impairment reported above for the SU-DNMS task, the inability of animals with Neo-PRh lesions to solve the OBJ-SO task could also be due to inability to suppress interference. The OBJ-SO task uses the same three stimuli from trial to trial, and across all daily sessions, resulting in high levels of interference. Thus, as reported above for the SU-DNMS, the severe impairment on the OBJ-SO task after Neo-PRh lesions could be due either to an inability to monitor information in WM and/or to an inability to resolve interference.

To distinguish between these alternative interpretations, the animals were tested in the SOMT, a WM task that requires the ability to monitor the sequence of object presentations but uses novel objects in each trial. In the SOMT, use of trial-unique stimuli was intended to minimize the impact of interference, and so performance should depend only on the ability to monitor the temporal order of stimuli. Neo-PRh monkeys acquired the SOMT rules similarly to controls, requiring approximately the same number of sessions at each learning stage. During Probe trials, Neo-PRh, and Neo-C monkeys made similar numbers of correct choices for temporal judgments between Object 1 and Object 4 as they did for temporal judgments between Object 2 and Object 3, resulting in roughly equivalent Inner:Outer Ratio scores. Thus, measured with SOMT, neonatal PRh lesion appears to spare the ability to monitor items in WM. Therefore, the severe impairments of the same monkeys in OBJ-SO are likely to be caused by impairment in cognitive processes other than WM. Indeed, the increase in perseverative errors found in animals with Neo-PRh lesions while performing WM tasks with high proactive interference may have instead been caused by a lack of impulse control and/or impaired behavioral flexibility.

### Comparison with the neonatal hippocampal lesions (Neo-H)

The pattern of deficits in the three working memory tasks after the Neo-PRh lesions contrasted with those reported after the Neo-H lesions (Heuer and Bachevalier, 2011, 2013). Unlike Neo-PRh lesions, Neo-H lesions did not impact the ability to maintain information in memory but resulted in severe impairment in both tasks measuring monitoring WM processes. Taken together, these data indicate that the perirhinal cortex and the hippocampus play different roles in supporting the development of WM processes; i.e., the hippocampus supporting monitoring WM processes whereas the perirhinal resolving proactive interference.

### Conclusions

The present results suggest that the perirhinal cortex may be particularly important to resolve interference. Yet, it is not clear whether the deficits resulted from direct damage to the PRh or from downstream effects of the neonatal PRh lesions on the normal maturation of other neural structures, especially those with protracted anatomical and functional development, such as the PFC (Fuster, 2002; Overman et al., 2004; Conklin et al., 2007; Kolb et al., 2010; Perlman et al., 2015). Developmental studies in rodents (Tseng et al., 2009) and monkeys (Bertolino et al., 1997; Chlan-Fourney et al., 2000; Meng et al., 2013) have already demonstrated significant morphological and neurochemical changes in the lateral PFC as a result of early damage to the MTL structures. Given that the lateral PFC is critical for performance on WM tasks, the WM deficits after the neonatal PRh lesions may have resulted from maldevelopment of the PFC following disruption of inputs it receives from the PRh rather than damage to PRh *per se*. Disentangling these alternative interpretations will require the replication of the current experiments in a group of monkeys that will have received the same PRh lesions in adulthood.

## Author contributions

Data acquisition by AW and RN. Experimental design, data analysis, and manuscript preparation by AW, RN, and JB.

## Funding

This work was supported by the National Institute of Mental Health (MH-58846 to JB and T32-HD071845 to AW), the National Science Foundation (NSF-GRFP DGE-1444932 to AW), and the National Center for Research Resources P51RR165, currently supported by the Office of Research Infrastructure Programs/OD P51OD11132.

### Conflict of interest statement

The authors declare that the research was conducted in the absence of any commercial or financial relationships that could be construed as a potential conflict of interest.

## References

[B1] Aggleton J. Hunt P. Rawlins J. (1986). The effects of hippocampal lesions upon spatial and non-spatial tests of working memory. Behav. Brain Res. 19, 133–146. 10.1016/0166-4328(86)90011-2 3964405

[B2] Bachevalier J. Wright A. Katz J. (2013). Serial position functions following selective hippocampal lesions in monkeys: effects of delays and interference. Behav. Process. 93, 155–166. 10.1016/j.beproc.2012.11.012 23246643 PMC3684055

[B3] Baxter M. Browning P. Mitchell A. (2008). Perseverative interference with object-in-place scene learning in rhesus monkeys with bilateral ablation of ventrolateral prefrontal cortex. Learn. Mem. 15, 126–132. 10.1101/lm.804508 18299439 PMC2275654

[B4] Baxter M. G. Gaffan D. Kyriazis D. A. (2009). Ventrolateral prefrontal cortex is required for performance of a strategy implementation task but not reinforcer devaluation effects in rhesus monkeys. Eur. J. Neurosci. 29, 2049–2059. 10.1111/j.1460-9568.2009.06740.x 19453635 PMC2688497

[B5] Bertolino A. Saunders R. Mattay V. Bachevalier J. Frank J. Weinberger D. (1997). Altered development of prefrontal neurons in rhesus monkeys with neonatal mesial temporo-limbic lesions: a proton magnetic resonance spectroscopic imaging study. Cereb. Cortex 7, 740–748. 10.1093/cercor/7.8.740 9408038

[B6] Brown M. Aggleton J. (2001). Recognition memory: what are the roles of the perirhinal cortex and hippocampus? Nat. Rev. Neurosci. 2, 51–61. 10.1038/35049064 11253359

[B7] Butter C. M. (1969). Perseveration in extinction and in discrimination reversal tasks following selective frontal ablations in *Macaca mulatta*. Physiol. Behav. 4, 163–171.

[B8] Butterly D. Petroccione M. Smith D. (2012). Hippocampal context processing is critical for interference free recall of odor memories in rats. Hippocampus 22, 906–913. 10.1002/hipo.20953 21542056 PMC3151480

[B9] Cannon T. Glahn D. Kim J. Van Erp T. Karlsgodt K. Cohen M.. (2005). Dorsolateral prefrontal cortex activity during maintenance and manipulation of information in working memory in patients with schizophrenia. Arch. Gen. Psychiatry 62, 1071–1080. 10.1001/archpsyc.62.10.1071 16203952

[B10] Cavada C. Tejedor J. Cruz-Rizzolo R. J. Reinoso-Suárez F. (2000). The anatomical connections of the macaque monkey orbitofrontal cortex. A review. Cereb. Cortex 10, 220–242. 10.1093/cercor/10.3.220 10731218

[B11] Chlan-Fourney J. Webster M. J. Felleman D. J. Bachevalier J. (2000). Neonatal medial temporal lobe lesions alter the distribution of tyrosine hydroxylase immunoreactive varicosities in the macaque prefrontal cortex, in Abstract Presented at the Society for Neuroscience Meeting (Washington, DC).

[B12] Cohen J. (1973). Eta-squared and partial eta-squared in fixed factor ANOVA designs. Educ. Psychol. Meas. 33, 107–112. 10.1177/001316447303300111

[B13] Conklin H. M. Luciana M. Hooper C. J. Yarger R. S. (2007). Working memory performance in typically developing children and adolescents: behavioral evidence of protracted frontal lobe development. Dev. Neuropsychol. 31, 103–128. 10.1207/s15326942dn3101_6 17305440

[B14] Constantinidis C. Procyk E. (2004). The primate working memory networks. Cogn. Affect. Behav. Neurosci. 4, 444–465. 10.3758/CABN.4.4.444 15849890 PMC3885185

[B15] Croxson P. L. Johansen-Berg H. Behrens T. E. Robson M. D. Pinsk M. A. Gross C. G.. (2005). Quantitative investigation of connections of the prefrontal cortex in the human and macaque using probabilistic diffusion tractography. J. Neurosci. 25, 8854–8866. 10.1523/JNEUROSCI.1311-05.2005 16192375 PMC6725599

[B16] D'Esposito M. Postle B. Ballard D. Lease J. (1999). Maintenance versus manipulation of information held in working memory: an event-related fMRI study. Brain Cogn. 41, 6686. 10536086 10.1006/brcg.1999.1096

[B17] Davachi L. Goldman-Rakic P. S. (2001). Primate rhinal cortex participates in both visual recognition and working memory tasks: functional mapping with 2-DG. J. Neurophysiol. 85, 2590–2601. 11387403 10.1152/jn.2001.85.6.2590

[B18] Diamond A. Zola-Morgan S. Squire L. R. (1989). Successful performance by monkeys with lesions of the hippocampal formation on AB and object retrieval, two tasks that mark developmental changes in human infants. Behav. Neurosci. 103, 526–537. 2736067 10.1037//0735-7044.103.3.526

[B19] Dias R. Robbins T. W. Roberts A. C. (1996). Dissociation in prefrontal cortex of affective and attentional shifts. Nature 380, 69–72. 10.1038/380069a0 8598908

[B20] Eacott M. Gaffan D. Murray E. (1994). Preserved recognition memory for small sets, and impaired stimulus identification for large sets, following rhinal cortex ablations in monkeys. Eur. J. Neurosci. 6, 1466–1478. 10.1111/j.1460-9568.1994.tb01008.x 8000570

[B21] Fuster J. M. (2002). Frontal lobe and cognitive development. J. Neurocytol. 31, 373–385. 10.1023/A:1024190429920 12815254

[B22] Goldman-Rakic P. S. Selemon L. D. Schwartz M. L. (1984). Dual pathways connecting the dorsolateral prefrontal cortex with the hippocampal formation and parahippocampal cortex in the rhesus monkey. Neuroscience 12, 719–743. 10.1016/0306-4522(84)90166-0 6472617

[B23] Goursaud A. Bachevalier J. (2007). Social attachment in juvenile monkeys with neonatal lesion of the hippocampus, amygdala and orbital frontal cortex. Behav. Brain Res. 176, 75–93. 10.1016/j.bbr.2006.09.020 17084912

[B24] Heuer E. Bachevalier J. (2011). Neonatal hippocampal lesions in rhesus macaques alter the monitoring, but not maintenance, of information in working memory. Behav. Neurosci. 125, 859–870. 10.1037/a0025541 21928873 PMC3226899

[B25] Heuer E. Bachevalier J. (2013). Working memory for temporal order is impaired after selective neonatal hippocampal lesions in adult rhesus macaques. Behav. Brain Res. 239, 55–62. 10.1016/j.bbr.2012.10.043 23137699 PMC3804023

[B26] Keppel G. Wickens T. D. (2004). Design and Analysis, 4th Edn. Upper Saddle River, NJ: Pearson.

[B27] Kimble D. P. Pribram K. H. (1963). Hippocampectomy and behavior sequences. Science 139, 824–825. 10.1126/science.139.3557.824 14032718

[B28] Kolb B. Halliwell C. Gibb R. (2010). Factors influencing neocortical development in the normal and injured brain, in Oxford Handbook of Developmental Behavioral Neuroscience, eds Blumberg M. S. Freeman J. H. Robinson S. R. (New York, NY: Oxford University Press), 375–388.

[B29] Kowalska D. M. Bachevalier J. Mishkin M. (1991). The role of the inferior prefrontal convexity in performance of delayed nonmatching-to-sample. Neuropsychologia 29, 583–600. 10.1016/0028-3932(91)90012-W 1944863

[B30] Lavenex P. Suzuki W. Amaral D. (2002). Perirhinal and parahippocampal cortices of the macaque monkey: projections to the neocortex. J. Comp. Neurol. 447, 394–420. 10.1002/cne.10243 11992524

[B31] Lavenex P. Suzuki W. Amaral D. (2004). Perirhinal and parahippocampal cortices of the macaque monkey: intrinsic projections and interconnections. J. Comp. Neurol. 472, 371–394. 10.1002/cne.20079 15065131

[B32] Lee A. Buckley M. Gaffan D. Emery T. Hodges J. Graham K. (2006). Differentiating the roles of the hippocampus and perirhinal cortex in processes beyond long-term declarative memory: a double dissociation in dementia. J. Neurosci. 26, 5198–5203. 10.1523/JNEUROSCI.3157-05.2006 16687511 PMC6674247

[B33] Lehky S. Tanaka K. (2007). Enhancement of object representations in primate perirhinal cortex during a visual working-memory task. J. Neurophysiol. 97, 1298–1310. 10.1152/jn.00167.2006 17108097

[B34] Levine T. Hullett C. (2002). Eta Squared, Partial Eta Squared, and misreporting of effect size in communication research. Hum. Commun. Res. 28, 612–625. 10.1111/j.1468-2958.2002.tb00828.x

[B35] Libby L. Ekstrom A. Ragland J. Ranganath C. (2012). Differential connectivity of perirhinal and parahippocampal cortices within human hippocampal subregions revealed by high-resolution functional imaging. J. Neurosci. 32, 6550–6560. 10.1523/JNEUROSCI.3711-11.2012 22573677 PMC3374643

[B36] Malkova L. Alvarado M. C. Bachevalier J. (2015). Effects of separate or combined neonatal damage to the orbital frontal cortes and the inferior convexity on object recognition memory in monkeys. Cereb. Cortex. [Epub ahead of print]. 10.1093/cercor/bhu227 25260702 PMC4712798

[B37] Málková L. Lex C. K. Mishkin M. Saunders R. C. (2001). MRIâĂŘbased evaluation of locus and extent of neurotoxic lesions in monkeys. Hippocampus 11, 361–370. 10.1002/hipo.1050 11530840

[B38] Meng Y. Li L. Hu X. Bachevalier J. Payne C. Zhang X. (2013). Differential alterations of dorsolateral and ventrolateral prefrontal cortex in adult macaques with neonatal hippocampal lesion: A diffusion Tensor Imaging study, in Abstract presented at International Society for Magnetic Resonance in Medicine (Salt Lake City, UT).

[B39] Meunier M. Bachevalier J. Mishkin M. (1997). Effects of orbital frontal and anterior cingulate lesions on object and spatial memory in rhesus monkeys. Neuropsychologia 35, 999–1015. 10.1016/S0028-3932(97)00027-4 9226661

[B40] Mishkin M. Manning F. J. (1978). Non-spatial memory after selective prefrontal lesions in monkeys. Brain Res. 143, 313–323. 10.1016/0006-8993(78)90571-1 415803

[B41] Mishkin M. Vest B. Waxler M. Rosvold H. E. (1969). A re-examination of the effects of frontal lesions on object alternation. Neuropsychologia 7, 357–363. 10.1016/0028-3932(69)90060-8

[B42] Morris R. Pandya D. N. Petrides M. (1999). Fiber system linking the mid-dorsolateral frontal cortex with the retrosplenial/presubicular region in the rhesus monkey. J. Comp. Neurol. 407, 183–192. 10213090 10.1002/(sici)1096-9861(19990503)407:2<183::aid-cne3>3.0.co;2-n

[B43] Murray E. A. Gaffan D. Mishkin M. (1993). Neural substrates of visual stimulus-stimulus association in rhesus monkeys. J. Neurosci. 13, 4549–4561 8410203 10.1523/JNEUROSCI.13-10-04549.1993 PMC6576366

[B44] National Research Council (US) (2011). Guide for the Care and Use of Laboratory Animals. 8th Edn Washington, DC: National Academies Press.

[B45] Nemanic S. Alvarado M. C. Price R. E. Jackson E. F. Bachevalier J. (2002). Assessment of locus and extent of neurotoxic lesions in monkeys using neuroimaging techniques: a replication. J. Neurosci. Methods 121, 199–209. 10.1016/S0165-0270(02)00264-9 12468009

[B46] Overman W. H. Frassrand K. Ansel S. Trawalter S. Bies B. Redmond A. (2004). Performance on the IOWA card task by adolescents and adults. Neuropsychologia 42, 1838–1851. 10.1016/j.neuropsychologia.2004.03.014 15351632

[B47] Owen A. M. Herrod N. J. Menon D. K. Clark J. C. Downey S. Carpenter A. T.. (1999). Redefining the functional organization of working memory processes within human lateral prefrontal cortex. Eur. J. Neurosci. 11, 567–574. 10.1046/j.1460-9568.1999.00449.x 10051756

[B48] Passingham R. (1975). Delayed matching after selective prefrontal lesions in monkeys (*Macaca mulatta*). Brain Res. 92, 89–102. 809096 10.1016/0006-8993(75)90529-6

[B49] Perlman S. B. Huppert T. J. Luna B. (2015). Functional near-infrared spectroscopy evidence for development of prefrontal engagement in working memory in early through middle childhood. Cereb. Cortex. [Epub ahead of print]. 10.1093/cercor/bhv139 26115660 PMC4869813

[B50] Petrides M. (1991). Functional specialization within the dorsolateral frontal cortex for serial order memory. Proc. R. Soc. B Biol. Sci. 246, 299–306. 10.1098/rspb.1991.0158 1686096

[B51] Petrides M. (1995). Impairments on nonspatial self-ordered and externally ordered working memory tasks after lesions of the mid-dorsal part of the lateral frontal cortex in the monkey. J. Neurosci. 15, 359–375. 7823141 10.1523/JNEUROSCI.15-01-00359.1995 PMC6578311

[B52] Petrides M. (2000). The role of the mid-dorsolateral prefrontal cortex in working memory, in Executive Control and the Frontal Lobe: Current Issues, eds Schneider W. X. Owen A. M. Duncan J. (Berlin; Heidelberg: Springer), 44–54. 10.1007/s002210000399 10933209

[B53] Petrides M. Pandya D. (2002). Comparative cytoarchitectonic analysis of the human and the macaque ventrolateral prefrontal cortex and corticocortical connection patterns in the monkey. Eur. J. Neurosci. 16, 291–310. 10.1046/j.1460-9568.2001.02090.x 12169111

[B54] Ranganath C. Cohen M. X. Dam C. D'Esposito M. (2004). Inferior temporal, prefrontal, and hippocampal contributions to visual working memory maintenance and associative memory retrieval. J. Neurosci. 24, 3917–3925. 10.1523/JNEUROSCI.5053-03.2004 15102907 PMC6729418

[B55] Raper J. Bachevalier J. Wallen K. Sanchez M. (2013). Neonatal amygdala lesions alter basal cortisol levels in infant rhesus monkeys. Psychoneuroendocrinology 38, 818–829. 10.1016/j.psyneuen.2012.09.004 23159012 PMC3582756

[B56] Rosnow R. Rosenthal R. (1996). Computing contrasts, effect sizes, and counternulls on other people's published data: general procedures for research consumers. Psychol. Methods 1:331 10.1037/1082-989X.1.4.331

[B57] Saunders R. Mishkin M. Aggleton J. (2005). Projections from the entorhinal cortex, perirhinal cortex, presubiculum, and parasubiculum to the medial thalamus in macaque monkeys: identifying different pathways using disconnection techniques. Exp. Brain Res. 167, 1–16. 10.1007/s00221-005-2361-3 16143859

[B58] Shapiro R. C. Olton D. S. (1994). Hippocampal function and interference, in Memory Systems, eds Schacter D. Tulving E. (London: MIT), 87–117.

[B59] Stern C. Sherman S. Kirchhoff B. Hasselmo M. (2001). Medial temporal and prefrontal contributions to working memory tasks with novel and familiar stimuli. Hippocampus 11, 337–346. 10.1002/hipo.1048 11530838

[B60] Suzuki W. A. Amaral D. G. (1994). Topographic organization of the reciprocal connections between the monkey entorhinal cortex and the perirhinal and parahippocampal cortices. J. Neurosci. 14, 1856–1877. 8126576 10.1523/JNEUROSCI.14-03-01856.1994 PMC6577578

[B61] Tseng K. Chambers R. Lipska B. (2009). The neonatal ventral hippocampal lesion as a heuristic neurodevelopmental model of schizophrenia. Behav. Brain Res. 204, 295–305. 10.1016/j.bbr.2008.11.039 19100784 PMC2735579

[B62] Ungerleider L. G. Courtney S. M. Haxby J. V. (1998). A neural system for human visual working memory. Proc. Natl. Acad. Sci. U.S.A. 95, 883–890. 10.1073/pnas.95.3.883 9448255 PMC33812

[B63] Warburton E. Brown M. (2010). Findings from animals concerning when interactions between perirhinal cortex, hippocampus and medial prefrontal cortex are necessary for recognition memory. Neuropsychologia 48, 2262–2272. 10.1016/j.neuropsychologia.2009.12.022 20026141

[B64] Warren D. E. Duff M. C. Jensen U. Tranel D. Cohen N. J. (2012). Hiding in plain view: lesions of the medial temporal lobe impair online representation. Hippocampus. 22, 1577–1588. 10.1002/hipo.21000 22180166 PMC3319639

[B65] Weiss A. R. Bachevalier J. (2016). Object and spatial memory after neonatal perirhinal lesions in monkeys. Behav. Brain Res. 298, 210–217. 10.1016/j.bbr.2015.11.010 26593109 PMC4688056

[B66] Zeamer A. Richardson R. L. Weiss A. R. Bachevalier J. (2015). The development of object recognition memory in rhesus macaques with neonatal lesions of the perirhinal cortex. Dev. Cogn. Neurosci. 11, 31–41. 10.1016/j.dcn.2014.07.002 25096364 PMC4302071

