# Supplementary material for: Neonatal Perirhinal Lesions in Rhesus Macaques Alter Performance on Working Memory Tasks with High Proactive Interference
Source: Front Syst Neurosci. 2016 Jan 5;9:179. doi: 10.3389/fnsys.2015.00179 (PMC4700260; doi:10.3389/fnsys.2015.00179)
Supplement: Supplementary file 1 [file Table1.DOCX]

**Supplementary Material**

**Table 1: Correlations between lesion extent and scores on cognitive tasks.**

Correlations between extent of neonatal PRh lesions or unintended damage to adjacent ERh areas and scores on the three tasks were performed with Pearson correlation. None of the correlations between the average or weighted average of bilateral PRh or ERh damage reached significance. There were, however, two significant negative correlations between the extent of right-side perirhinal damage and sessions to criterion for two of the 4-Object SOMT problems: 1v3 and 2v3. (L% = percent damage to left hemisphere; R% = percent damage to right hemisphere; X% = average damage to both hemispheres; W% = weighted damage to both hemispheres [W% = (L% X R%)/100]).

|  | | | | | | | | | |
| --- | --- | --- | --- | --- | --- | --- | --- | --- | --- |
|  | | **L% PRh** | **R% PRh** | **X% PRh** | **W% PRh** | **L% ERh** | **R% ERh** | **X% ERh** | **W% ERh** |
| **SUDNMS**  **Errors_5s** | R | -.153 | .397 | .024 | .017 | -.490 | -.734 | -.744 | -.741 |
|  | Sig. (2-tailed) | .772 | .436 | .965 | .975 | .324 | .097 | .090 | .092 |
| **SUDNMS**  **Errors_30s** | R | -.389 | -.315 | -.460 | -.455 | -.371 | -.059 | -.224 | -.194 |
|  | Sig. (2-tailed) | .446 | .543 | .358 | .364 | .469 | .912 | .670 | .712 |
| **SUDNMS**  **ErrorRatio_5s** | R | .172 | .054 | .160 | .138 | .156 | -.444 | -.225 | -.101 |
|  | Sig. (2-tailed) | .744 | .918 | .762 | .794 | .767 | .377 | .669 | .849 |
| **SUDNMS**  **ErrorRatio_30s** | R | -.472 | -.241 | -.464 | -.485 | -.200 | .180 | .025 | .238 |
|  | Sig. (2-tailed) | .345 | .645 | .354 | .330 | .704 | .733 | .962 | .649 |
| **OBJSO**  **Sessions to Criterion** | R | -.176 | .728 | .187 | .189 | -.726 | -.318 | -.579 | -.640 |
|  | Sig. (2-tailed) | .739 | .101 | .723 | .721 | .102 | .539 | .229 | .171 |
| **OBJSO**  **PrimaryErrors_Trial2** | R | .057 | .393 | .200 | .214 | -.527 | -.576 | -.656 | -.793 |
|  | Sig. (2-tailed) | .915 | .440 | .704 | .684 | .282 | .232 | .157 | .060 |
| **OBJSO**  **PrimaryErrors_Trial3** | R | -.083 | .572 | .186 | .198 | -.582 | -.216 | -.438 | -.556 |
|  | Sig. (2-tailed) | .876 | .236 | .724 | .707 | .225 | .681 | .385 | .251 |
| **OBJSO**  **PersevErrors_Trial2** | R | -.061 | .089 | -.042 | -.020 | -.448 | -.423 | -.512 | -.674 |
|  | Sig. (2-tailed) | .909 | .867 | .937 | .970 | .374 | .404 | .300 | .142 |
| **OBJSO**  **PersevErrors_Trial3** | R | -.046 | .602 | .234 | .246 | -.456 | -.024 | -.244 | -.375 |
|  | Sig. (2-tailed) | .931 | .206 | .655 | .638 | .363 | .965 | .641 | .464 |
| **SOMT3**  **1v3** | R | .126 | .208 | .205 | .213 | -.251 | -.125 | -.211 | -.280 |
|  | Sig. (2-tailed) | .812 | .693 | .696 | .685 | .631 | .813 | .688 | .591 |
| **SOMT3**  **1v2** | R | .385 | -.506 | .063 | .061 | .593 | -.251 | .124 | .141 |
|  | Sig. (2-tailed) | .451 | .305 | .905 | .908 | .215 | .631 | .815 | .790 |
| **SOMT3**  **2v3** | R | -.499 | -.116 | -.464 | -.460 | -.107 | .433 | .242 | .253 |
|  | Sig. (2-tailed) | .314 | .826 | .354 | .359 | .840 | .391 | .644 | .629 |
| **SOMT4**  **1v4** | R | -.249 | -.299 | -.388 | -.365 | -.194 | -.241 | -.261 | -.399 |
|  | Sig. (2-tailed) | .634 | .565 | .447 | .477 | .713 | .645 | .617 | .433 |
| **SOMT4**  **1v3** | R | -.155 | -.910 | -.548 | -.547 | .422 | .211 | .355 | .429 |
|  | Sig. (2-tailed) | .769 | .012* | .260 | .262 | .404 | .688 | .489 | .396 |
| **SOMT4**  **1v2** | R | -.653 | -.176 | -.643 | -.632 | -.478 | -.046 | -.268 | -.303 |
|  | Sig. (2-tailed) | .160 | .739 | .169 | .179 | .338 | .931 | .607 | .559 |
| **SOMT4**  **2v4** | R | .207 | .542 | .435 | .439 | .095 | .450 | .352 | .265 |
|  | Sig. (2-tailed) | .694 | .267 | .389 | .383 | .858 | .371 | .493 | .612 |
| **SOMT4**  **3v4** | R | .315 | .460 | .504 | .507 | .232 | .623 | .539 | .483 |
|  | Sig. (2-tailed) | .543 | .359 | .307 | .305 | .658 | .187 | .270 | .332 |
| **SOMT4**  **2v3** | R | -.059 | -.864 | -.447 | -.438 | .365 | .205 | .323 | .340 |
|  | Sig. (2-tailed) | .912 | .026* | .374 | .385 | .477 | .696 | .533 | .509 |
| **SOMTProbe**  **InnerOuterRatio** | R | -.641 | -.430 | -.722 | -.730 | -.076 | .280 | .155 | .280 |
|  | Sig. (2-tailed) | .171 | .395 | .105 | .100 | .887 | .592 | .770 | .591 |
